# Influence of pH, Heat Treatment of Inoculum, and Selenium Oxyanions on Concomitant Selenium Bioremediation and Volatile Fatty Acid Production from Food Waste

**DOI:** 10.1021/acsomega.2c06459

**Published:** 2023-09-14

**Authors:** Mohanakrishnan Logan, Fengyi Zhu, Piet N. L. Lens, Zeynep Cetecioglu

**Affiliations:** † Department of Chemical Engineering, School of Engineering Sciences in Chemistry, Biotechnology and Health, KTH Royal Institute of Technology, Stockholm SE 100 44, Sweden; ‡ Department of Microbiology, School of Natural Sciences and Ryan Institute, National University of Ireland, University Road, Galway H91 TK33, Ireland; § Department of Industrial Biotechnology, School of Engineering Sciences in Chemistry, Biotechnology and Health, KTH Royal Institute of Technology, Stockholm SE 106 91, Sweden

## Abstract

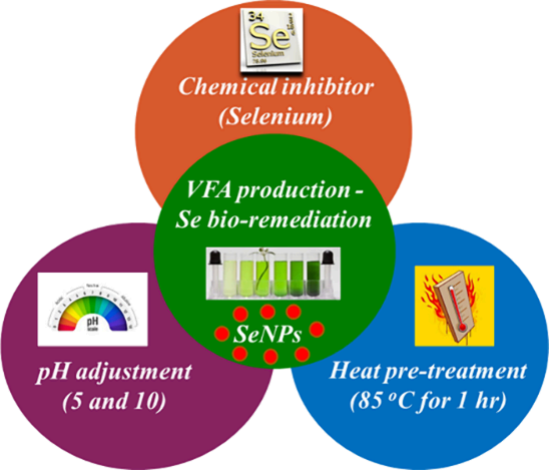

Developing novel strategies to enhance volatile fatty acid (VFA) yield from abundant waste resources is imperative to improve the competitiveness of biobased VFAs over petrochemical-based VFAs. This study hypothesized to improve the VFA yield from food waste via three strategies, viz., pH adjustment (5 and 10), supplementation of selenium (Se) oxyanions, and heat treatment of the inoculum (at 85 °C for 1 h). The highest VFA yield of 0.516 g COD/g VS was achieved at alkaline pH, which was 45% higher than the maximum VFA production at acidic pH. Heat treatment resulted in VFA accumulation after day 10 upon alkaline pretreatment. Se oxyanions acted as chemical inhibitors to improve the VFA yield at pH 10 with non-heat-treated inoculum (NHT). Acetic and propionic acid production was dominant at alkaline pH (NHT); however, the VFA composition diversified under the other tested conditions. More than 95% Se removal was achieved on day 1 under all the conditions tested. However, the heat treatment was detrimental for selenate reduction, with less than 15% Se removal after 20 days. Biosynthesized Se nanoparticles were confirmed by transmission and scanning electron microscopy and and energy dispersive X-ray analyses. The heat treatment inhibited the presence of nonsporulating bacteria and methanogenic archaea (*Methanobacteriaceae*). High-throughput sequencing also revealed higher relative abundances of the bacterial families (such as *Clostridiaceae*, *Bacteroidaceae*, and *Prevotellaceae*) that are capable of VFA production and/or selenium reduction.

## 1 Introduction

Biobased circular economy regards organic waste as a potential resource that can be utilized to recover valuable fuels, nutrients, and chemicals. Resource recovery from waste streams helps to achieve the ambitious target of net-zero carbon emissions by 2050 as well as the United Nations Sustainable Development Goals.^1^ With one-third of food produced ending up as waste, food waste alone generates about 8–10% of the global greenhouse gas emissions.^2^ Notably, Europe dominated the food waste management market and accounted for a 32.4% share of the global revenue in 2019 owing to reasons such as excessive shopping, improper food management, overproduction, and negligence for wasted food.^3^

Methane, which is the final end product of anaerobic digestion (AD), has a low commercial value of less than € 0.1/m^3^.^4^ Further, challenges from fugitive methane emissions and affordable solar and wind energy in recent times raise concerns over the long-term sustainability of AD technology for renewable energy production. On the other hand, volatile fatty acids (VFAs) are the intermediate products of anaerobic treatment and have high economic value and marketability. The market demand for VFAs in 2020 was 18,500 kilotons and is projected to increase at a growth rate of 3% per year.^1^ Recovery of VFAs helps carbon-intensive industries (such as agri-food, dairy, pharmaceutical, and manufacturing) and the energy sector to decarbonize their production, decrease the environmental impact, and generate revenue. Depending upon their molecular structure, VFAs have a 4 to 25 times higher market value than biogas.^5^ A wide range of waste, including food waste, can be stabilized by VFA production from them.^6^ These VFAs derived from anaerobic fermentation can be transformed into electricity by microbial fuel cells.^7^ Alternatively, VFAs can also serve as an excellent carbon source for the production of polyhydroxyalkanoates or bioplastics.^8,9^ The use of VFA-rich fermentation liquids is a sustainable carbon source for biological removal of nutrients such as phosphorus and nitrogen.^10^ Research on production of single-cell protein to meet the global food demand using VFAs is also gaining interest.^11^

Optimizing the operational parameters, such as pH or adding chemicals, can result in higher VFA yields. Chemicals such as 2-bromoethanesulfonic acid (BES) have been used as an inhibitor to suppress methanogenesis, resulting in improved VFA yield by preventing their consumption.^12^ However, the high cost of BES (∼€900/kg) necessitates identifying cheap chemical inhibitors, especially from waste streams. Although acidic pH was adopted conventionally for acidogenesis, few studies have successfully demonstrated that an alkaline pH can vastly increase VFA productivity.^13^ Similarly, pretreating inoculum by temperature shocks could limit the growth of homoacetogenic bacteria and methanogenic archaea.^14^ However, there have been very few studies showing improved VFA production due to heat treatment of the inoculum. Therefore, this study investigates enhancing VFA accumulation from food waste with heat-treated inoculum.

On the other hand, selenium (Se) wastewaters are of huge environmental concern owing to their acute and chronic toxicity.^15^ Anaerobic treatment of Se-laden wastewater (with an organic cosubstrate) offers the possibility to recover VFAs as well as to convert soluble Se oxyanions to insoluble and less toxic elemental Se. Similarly, the Se nanoparticles synthesized find application in several industries including health care, metallurgy, glass, and chemical manufacturing.^16^ Our previous long-term continuous study established that 500 μM Se oxyanions caused methanogenic inhibition and VFA accumulation during AD of glycerol containing wastewater.^17^ However, the influence of Se on the amount and composition of VFA produced is not clearly understood. To the best of our knowledge, no studies have focused on concomitant VFA production and Se reduction using food waste as the carbon source. Microbial studies establishing the coexistence of fermentative as well as selenium reducing bacterial communities are also scarce.

Therefore, this study hypothesizes to enhance VFA production via three different strategies, viz., pH adjustment (acidic and alkaline), chemical methanogenic inhibitor, and heat treatment of the inoculum. The study will indicate the feasibility for continuous and simultaneous VFA and Se nanoparticle production for commercial applications. The results pave the way for the circular economy and energy and resource recovery in industries such as agri-food, mining, coal-fired power generation, and oil refineries that generate food waste or Se-rich wastewaters. Demonstration of biogenic Se nanoparticle synthesis and VFA production will also cater to the needs of a wide range of industries.

## 2 Materials and Methods

### 2.1 Substrate and Inoculum

Food waste collected from a pilot plant established at a wastewater treatment facility of KTH (Hammerby Sjöstadverket, Stockholm, Sweden) was used as the substrate. The digested sludge was collected from a full-scale anaerobic digester at the Henriksdal wastewater treatment plant (Stockholm, Sweden) and was used as the inoculum. The characteristics of the food waste and digested sludge are presented in Table 1. Sodium selenate and sodium selenite salts were dissolved in distilled water to obtain stock solutions of selenate (SeO_4_^2–^) and selenite (SeO_3_^2–^).

**Table 1:** Characteristics of the Substrate and Inoculum Used in This Study

parameter	food waste	inoculum
TS (%)	9.37	2.61
VS (%)	8.10	1.66
pH	4.51	7.59
total COD (mg/L)	204700	51900
soluble COD (mg/L)	193933	49400

### 2.2 Experimental Setup

Using food waste as the substrate, the effects of pH, heat treatment of the inoculum, and speciation and concentration of selenium oxyanions on VFA production, selenium removal, and microbial community composition were investigated. The batch assays were conducted in 150 mL serum bottles (with 100 mL working volume) at 35 °C (mesophilic conditions) and 120 rpm mixing. A substrate to inoculum ratio of 2 g COD/g VS was adopted to maximize VFA production.^18^ The pH of the reactors was adjusted to 5.0 or 10.0 by using 1 M HCl or 1 M NaOH, respectively, to determine the pH effect on VFA production. SeO_4_^2–^ concentrations supplemented were as follows: 0 (control), 100 μM (14.30 mg/L), 300 μM (42.89 mg/L), and 500 μM (71.49 mg/L). Likewise, SeO_3_^2–^ concentrations were 0 (control), 100 μM (12.70 mg/L), 300 μM (38.09 mg/L), and 500 μM (63.49 mg/L). The results obtained at 100 and 300 μM are provided in the Supporting Information (Figures S1–S3). Each reactor was purged with nitrogen to ensure strict anaerobic conditions at the start of the experiment. Following this, the second phase of the experiment was conducted using the heat-treated inoculum, where the digested sludge was heated at 85 °C for 1 h in a muffle furnace and then used in batch reactors. A 20 day retention period was adopted, and the sacrificial bottles were set up to be taken down on days 1, 5, 10, 15, and 20. Each test condition was conducted in triplicate or duplicate for statistical analysis. Table S1 shows the experimental sets conducted in this study.

### 2.3 Analytical Methods

The pH was measured by a pH meter (Mettler Toledo FiveEasy pH bench meter, FE20). Total solids (TS, in %) and volatile solids (VS, in %) were determined by oven drying at 105 °C for 24 h and furnace drying at 550 °C for 2 h, respectively.^19^ Aliquots of the fermentation broth of each reactor were taken and centrifuged at 11,000 rpm for 3 min. The supernatant was filtered with 0.45 and 0.2 μm polypropylene filters to determine the soluble chemical oxygen demand (soluble COD) and VFA composition, respectively. The soluble COD was measured using a COD cuvette test (LCK 514 Hach Lange, Düsseldorf, Germany). The composition of the VFAs was determined using a high-performance liquid chromatograph (HPLC) (1260 Infinity II, Agilent, Santa Clara, USA) equipped with a Hi-Plex H (300 × 7.7 mm) column heated at 60 °C and a refractive index detector (RID) set at 55 °C. H_2_SO_4_ solution (5 mM) was used as the mobile phase at a flow rate of 0.7 mL/min and with a sample injection volume of 50 μL.^20^ The VFA concentrations measured are expressed as the COD equivalent. The COD conversion factors for acetic, propionic, butyric, valeric, isovaleric, caproic, and isocaproic acids amount to 1.067, 1.512, 1.816, 2.037, 2.037, 2.207, and 2.207, respectively.^21^

The liquid samples collected from the batch assays were analyzed for the Se oxyanion concentration. The collected liquid samples were centrifuged at 11,000 rpm for 15 min and filtered with 0.2 μm polypropylene filters to remove suspended cells and elemental selenium (Se^0^) particles. Total Se was quantified using an inductively coupled plasma–optical emission spectroscopy 5110 synchronous vertical dual view (ICP-OES 5110, Agilent Technologies, Santa Clara, USA).^22^ Transmission electron microscopy (TEM) and scanning electron microscopy (SEM) images of selenium nanoparticles were studied. The protocols followed for preparation, fixing, and imaging in TEM and SEM were described in detail by Florentino et al. (2020) and Tan and Lens (2021), respectively.^23,24^

### 2.4 Microbial Community Analyses

Total genomic DNA was extracted from the samples using a NuceloSpin Soil DNA kit (Macherey-Nagel, Germany) following the protocol recommended by the manufacturer. Polymerase chain reaction (PCR) amplifications of the DNA samples were performed using primers 515F-806R for amplifying the 16S rRNA genes using a PCR Master Mix (Taq DNA Polymerase, VWR International) in a thermal cycler (Techne Prime, Cole-Parmer, UK). The amplification conditions and library preparation for high-throughput sequencing were carried out with the method described by Owusu-Agyeman et al. (2022).^25^ Sequencing was done at the National Genomics Infrastructure (SciLife, Stockholm) using a MiSeq high-throughput sequencer (MSC 2.5.0.5/*RT*A 1.18.54). Bioinformatic analyses of the sequencing data were carried out using the QIIME2 and SILVA database.^26^ The Illumina MiSeq sequencing raw data were submitted to the National Center for Biotechnology Information (NCBI) as a BioProject submission with accession number PRJNA873110.

### 2.5 Calculations and Statistical Methods

The VFA production efficiency was calculated as the ratio of total VFA concentration to soluble COD concentration.^27^ Analysis of variance (ANOVA) was performed on the averaged VFA production data. Principal component analysis (PCA) was applied to the microbial diversity data. Pearson’s correlation analysis was conducted between the microbial diversity (relative abundance on family level), selenium, and VFA concentrations for different pH and heat treatment conditions. All analyses were conducted using IBM SPSS 16 and PAST 4.

## 3 Results

### 3.1 Effect of Acidic and Alkaline pH

**Figure 1:**
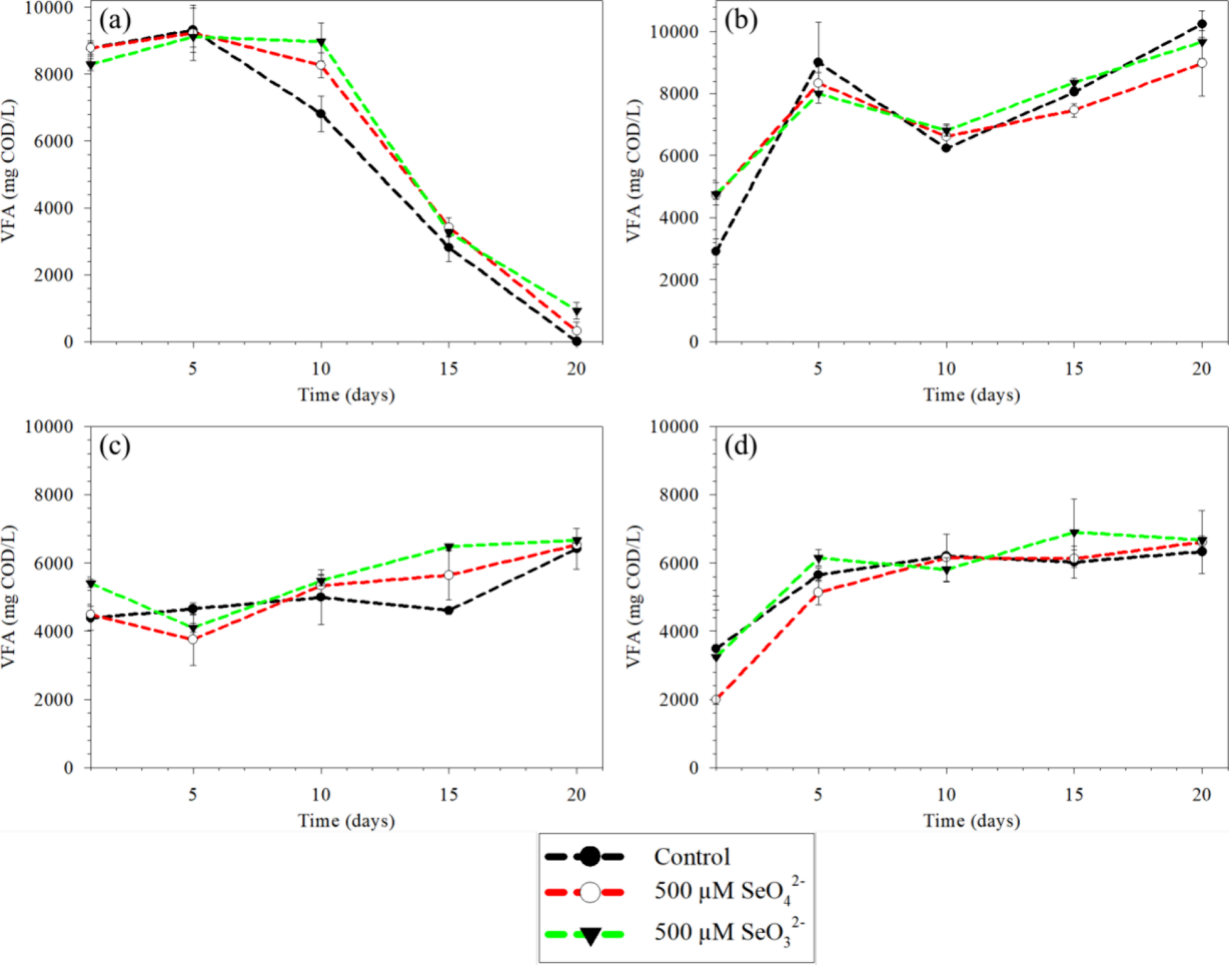
VFA profile at (a) pH 10, NHT; (b) pH 10, HT; (c) pH 5, NHT; and (d) pH 5, HT. NHT and HT stands for non-heat treated and heat treated inoculum, respectively.

**Figure 2:**
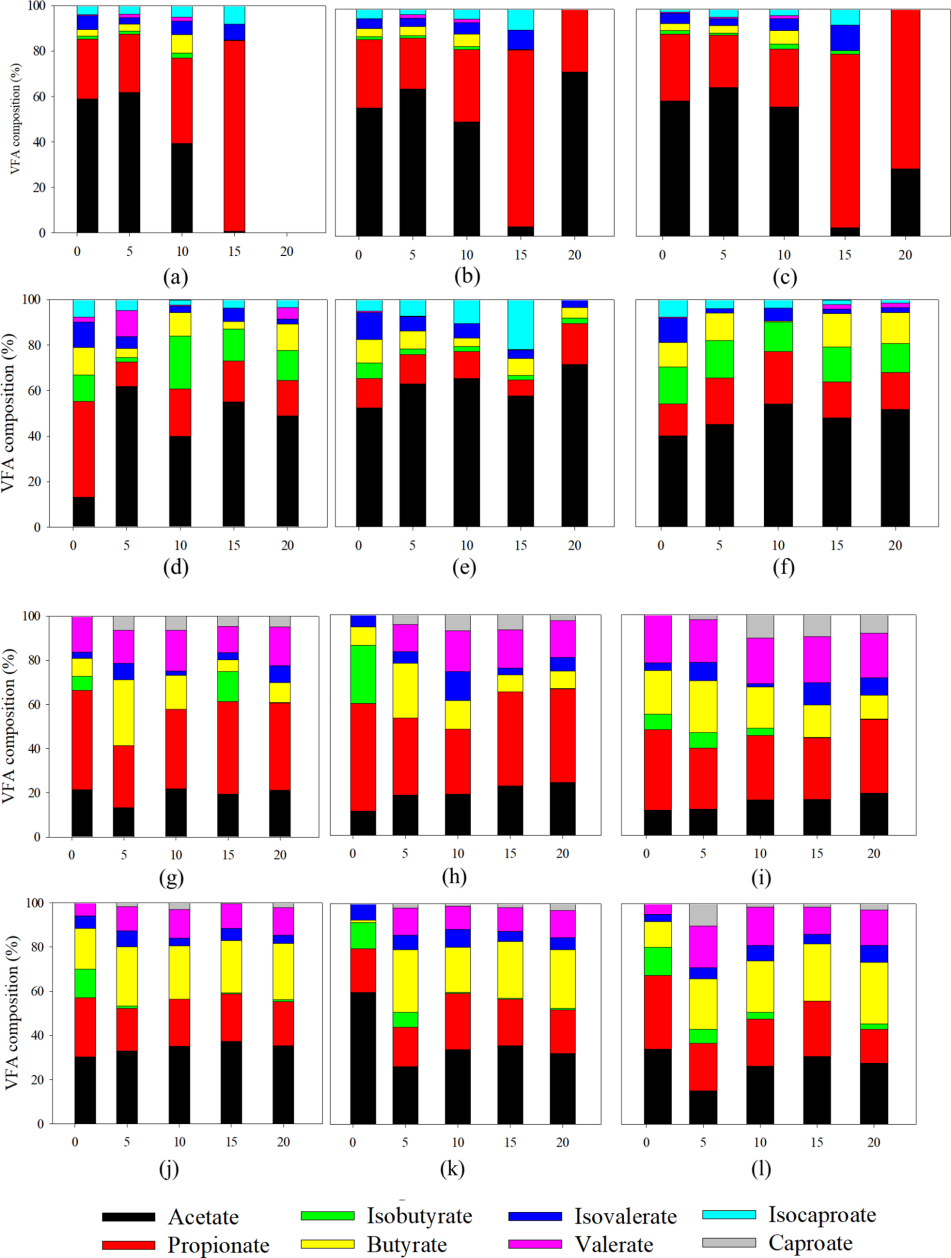
VFA composition at (a) pH 10, NHT, control; (b) pH 10, NHT, 500 μM SeO_4_^2–^; (c) pH 10, NHT, 500 μM SeO_3_^2–^; (d) pH 10, HT, control; (e) pH 10, HT, 500 μM SeO_4_^2–^; (f) pH 10, HT, 500 μM SeO_3_^2–^; (g) pH 5, NHT, control; (h) pH 5, NHT, 500 μM SeO_4_^2–^; (i) pH 5, NHT, 500 μM SeO_3_^2–^; (j) pH 5, HT, control; (k) pH 5, HT, 500 μM SeO_4_^2–^; and (l) pH 5, HT, 500 μM SeO_3_^2–^. For abbreviations, see Figure 1.

The initial VFA concentration introduced into the batch bottles from food waste is presented in Table S2. Figure 1 presents the profile of VFA production at different pH, speciation, and concentration of selenium with and without heat treatment. The VFA production over time decreased at pH 10 (Figure 1a). The maximum VFA production at pH 10 was 9315 (±652) mg COD/L on day 5. In contrast, the VFA production profile was in an increasing trend, with the maximum VFA production of 6416 (±591) mg COD/L on day 20 at pH 5. A higher VFA yield (by about 45.2%) was obtained in alkaline conditions at a shorter retention time when compared with the acidic environment (Figures 1a,c). At initial pH 10, the pH dropped to near neutral after 5 days and thereafter was in the range between 7 and 8 until the end of the incubation. However, the pH was below 5.6 throughout the experiment for the "initial pH 5" incubation (Figure S4). Figure 2 presents the VFA composition under the different test conditions. About 80% of the VFAs produced in alkaline conditions was dominated by acetate and propionate. On the contrary, a diverse composition of all VFAs except isocaproate was present under acidic conditions (Figure 2).

### 3.2 Effect of Inoculum Heat Treatment

Heat-treated inoculum resulted in VFA accumulation in an increasing trend to 10241 (±430) mg COD/L by day 20 at pH 10. In contrast, without heat treatment, complete VFA utilization resulted in the absence of VFAs on day 20 at pH 10 (Figure 1a). When compared with the effect of Se oxyanion supplementation (refer to Section 3.3), VFA accumulation was much more evident with the effect of heat treatment. Although heat treatment showed a clear improvement in the VFA yield at alkaline pH, there was no significant effect at acidic pH (Figure 1b–d). The VFA composition diversified because of heat treatment under alkaline conditions except caproic acid (Figure 2a–f). Heat treatment significantly changed the pH profile under alkaline conditions (Figure S4). pH gradually decreased to ∼8 only after day 15.

### 3.3 Effect of Selenium Oxyanions and Their Concomitant Reduction

Supplementation of selenium oxyanions prevented the complete VFA conversion and utilization at alkaline pH with non-heat-treated inoculum, which was remarkable on days 10, 15, and 20. In the presence of Se oxyanions, higher VFA yields were obtained on and after day 10 at pH 10. Selenite resulted in the highest VFA yield (8967 ± 569 mg COD/L) followed by selenate (8261 ± 371 mg COD/L) on day 10, which was about 32 and 21% higher than that of the control. The presence of Se oxyanions did not significantly increase the VFA yields under acidic conditions. Figures S1 and S2 show the VFA concentration and composition for 100 and 300 μM SeO_4_^2–^ and SeO_3_^2–^ at acidic and alkaline pH without heat treatment.

**Figure 3:**
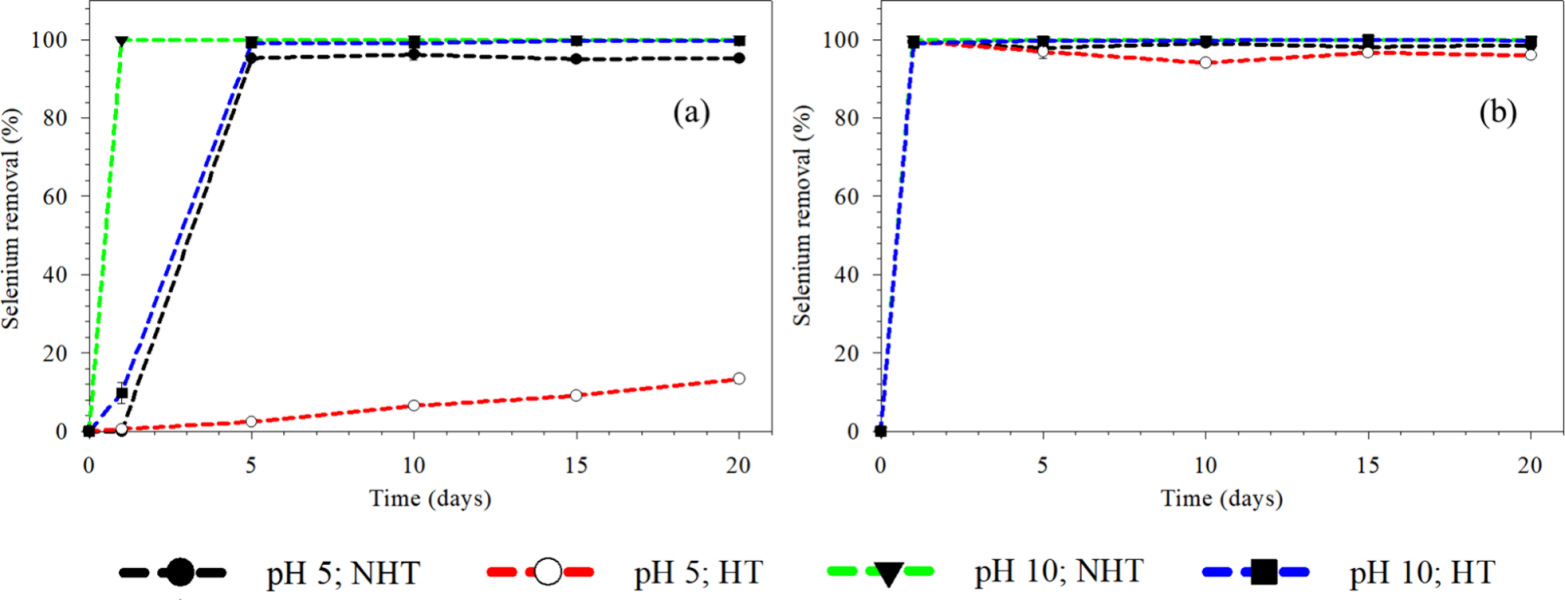
Selenium removal achieved from (a) 500 μM SeO_4_^2–^ and (b) 500 μM SeO_3_^2–^ at pH 5 and 10 with non-heat-treated (NHT) and heat-treated (HT) inoculum.

**Figure 4:**
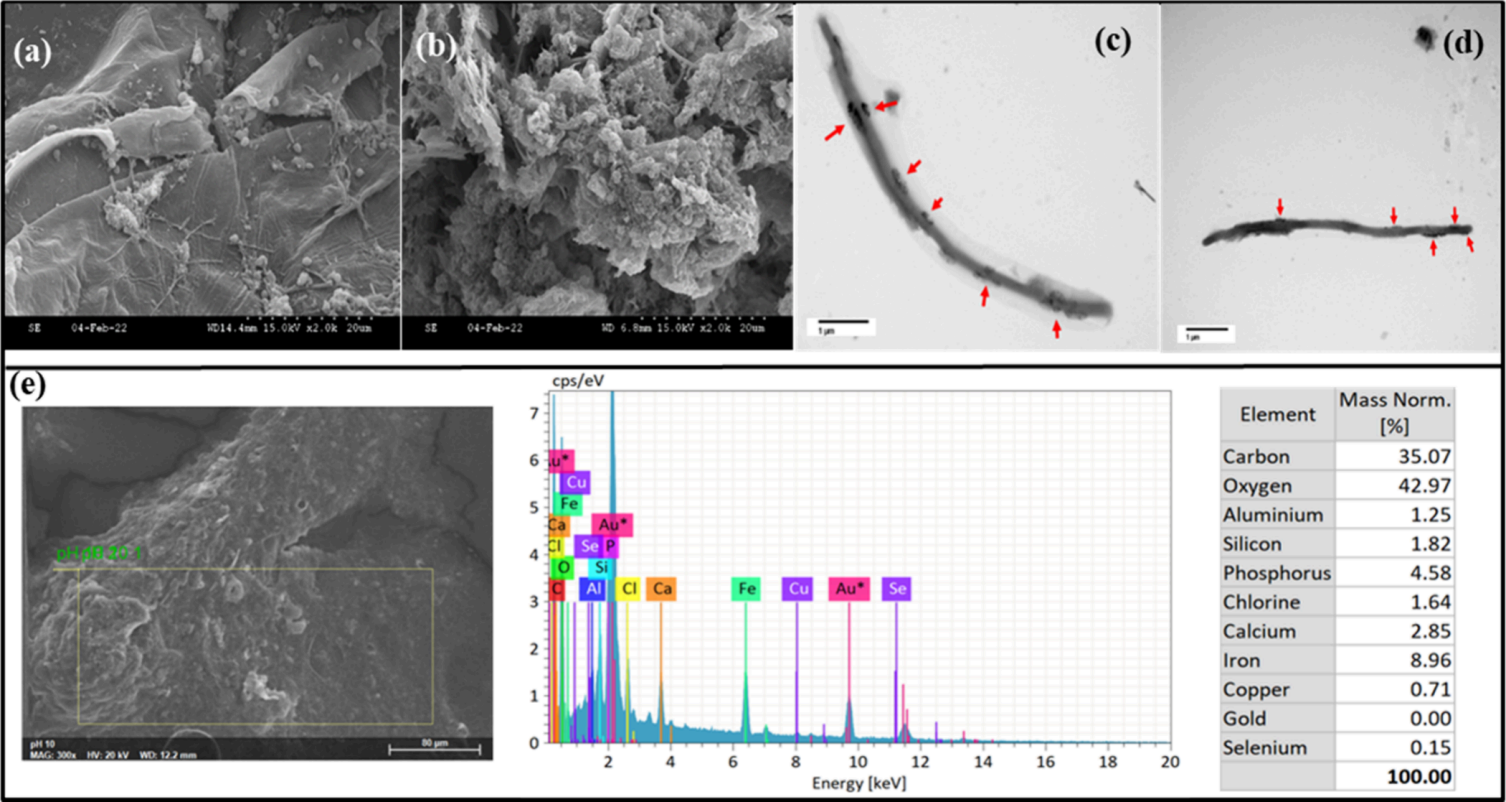
Electron microscopic images: SEM images (a, b) and TEM images (c, d) of the sludge taken from the end of the experiment (day 20) at pH 10 (NHT) to observe extracellular elemental selenium nanospheres deposited in the digested sludge flocs. SEM-EDX (e) confirms biosynthesized selenium nanoparticles deposited in the digested sludge flocs.

Se removal at acidic and alkaline pH with and without heat treatment is presented in Figure 3. More than 98% Se removal was observed at the end of day 1 in the presence of SeO_3_^2–^ under all tested conditions. On the other hand, above 95% Se removal was achieved on day 1 in the presence of SeO_4_^2–^ at alkaline-NHT; however, heat treatment delayed Se removal (95%) on day 5 at pH 10. At acidic pH (NHT), about 95% SeO_4_^2–^ removal efficiency was achieved on day 5. Interestingly, Se removal was less than 20% at acidic-NHT. Se removal at 100 and 300 μM SeO_4_^2–^ and SeO_3_^2–^ at pH 5 and 10 without heat treatment is presented in Figure S3. The reduction of Se oxyanions to elemental Se nanoparticles using food waste derived electron donors is supported by electron microscopic images. Figure 4a–d shows the SEM and TEM images of elemental Se nanoparticles deposited in the digested sludge flocs. SEM-EDX confirms that the nanoparticles observed in the electron microscopy images are indeed selenium nanoparticles, as shown in Figure 4e.

### 3.4 Effect of pH, Heat Treatment, and Se Oxyanions on Microbial Community

**Figure 5:**
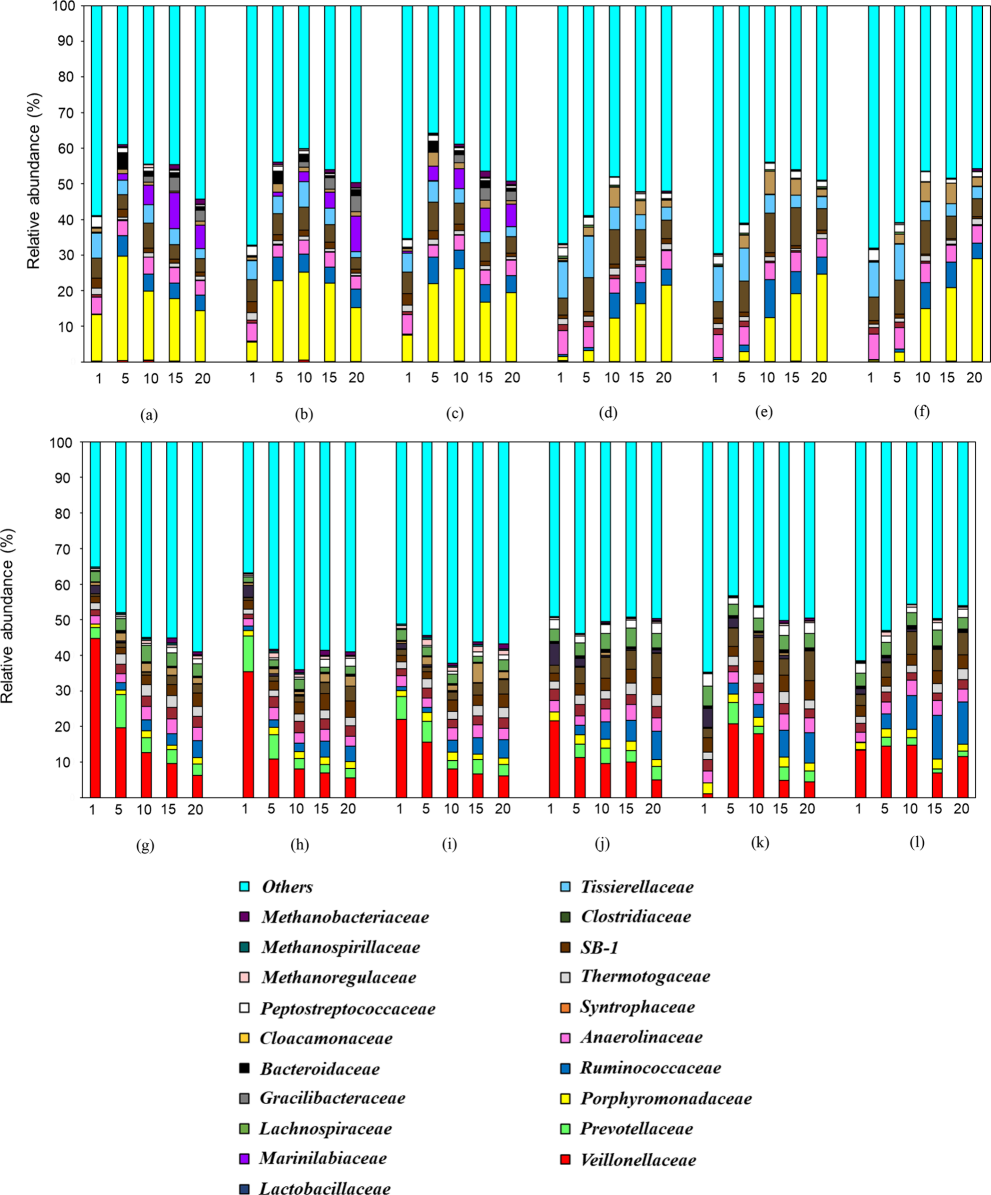
Relative abundance of the microbial communities at (a) pH 10, NHT, control; (b) pH 10, NHT, 500 μM SeO_4_^2–^; (c) pH 10, NHT, 500 μM SeO_3_^2–^; (d) pH 10, HT, control; (e) pH 10, HT, 500 μM SeO_4_^2–^; (f) pH 10, HT, 500 μM SeO_3_^2–^; (g) pH 5, NHT, control; (h) pH 5, NHT, 500 μM SeO_4_^2–^; (i) pH 5, NHT, 500 μM SeO_3_^2–^; (j) pH 5, HT, control; (k) pH 5, HT, 500 μM SeO_4_^2–^; and (l) pH 5, HT, 500 μM SeO_3_^2–^. Unclassified and RA < 2% at family level were grouped as “*Others*”. For abbreviations, see Figure 1.

The relative abundance (RA) of microbial communities was more dynamic at alkaline pH relative to that at acidic pH (Figure 5). The dominant taxon at the family level was *Porphyromonadaceae* followed by *Ruminococcaceae*, *Anaerolinaceae*, *Clostridiaceae*, and *Tissierellaceae* at alkaline pH. On the contrary, *Veillonellaceae* followed by *Prevotellaceae*, *Ruminococcaceae*, *Anaerolinaceae*, *SB-1*, *Clostridiaceae*, and *Cloacamonaceae* were the dominant taxa at acidic pH.

The effect of heat treatment was evident from the reduction of RA of a few microbial community members. The RA of *Porphyromonadaceae* was reduced from 13 to 1% on day 1 at pH 10 after heat treatment of the inoculum. However, their presence increased consistently to above 12% by the end of day 20 of incubation. Notably, heat treatment of the inoculum at an alkaline pH led to complete inhibition in the presence of *Marinilabiaceae* and a significant reduction in the presence of *Bacteroidaceae*. There was also a reduction in the presence of *Prevotellaceae* after heat treatment at acidic pH.

**Figure 6:**
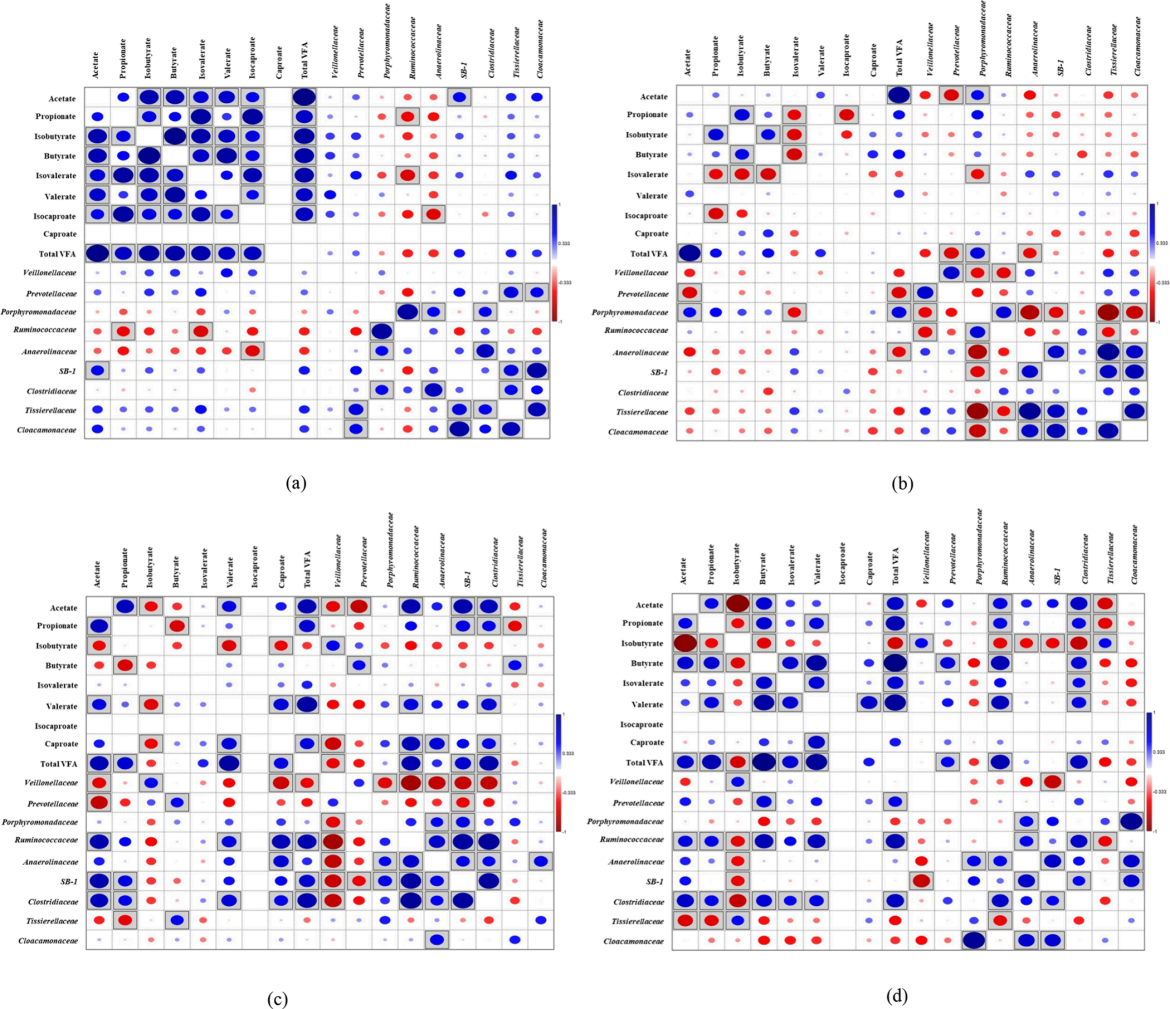
Pearson Correlation Analysis between bacterial families and volatile fatty acids for (a) pH 10, NHT; (b) pH 10, HT; (c) pH 5, NHT; and (d) pH 5, HT. The boxed circle indicates that the correlation is significant (*p* < 0.05). For abbreviations, see Figure 1.

The PCA results revealed robust clustering of the family members under acidic conditions, more than under alkaline conditions (Figure S5). The Pearson correlation analysis presented in Figure 6 shows that the *SB-1* family had a positive correlation with acetate production in non-heat treated conditions, whereas *Porphyromonadaceae* had a positive correlation with acetate and total VFA production after heat treatment in alkaline conditions. In acidic and non-heat treated conditions, positive correlations were observed with *Veillonellaceae* on isobutyrate; *Prevotellaceae* on butyrate; *Ruminococcaceae* on acetate, valerate, caproate, and total VFA; *Anaerolinaceae* on caproate; *Tissierellaceae* on butyrate; *SB-1* on acetate, propionate, and total VFA; and *Clostiridaceae* on acetate, propionate, valerate, caproate, and total VFA in acidic and non-heat treated conditions. On the other hand, positive correlations were observed with *Veillonellaceae* on isobutyrate; *Prevotellaceae* on butyrate and total VFA; *Ruminococcaceae* on acetate, propionate, butyrate, valerate, and total VFA; *Tissierellaceae* on isobutyrate; and *Clostiridaceae* on acetate, propionate, butyrate, isovalerate, valerate, and total VFA in acidic and non-heat treated conditions. Positive correlations between few bacterial families were observed under the tested conditions as depicted in Figure 6.

## 4 Discussion

### 4.1 Novel Strategies for Enhancing Volatile Fatty Acid Production Efficiency

#### 4.1.1 Improved VFA Yield at Alkaline pH

This study shows that initial alkaline conditions resulted in higher VFA production than that under acidic conditions (Figure 1a,c). This is in line with the findings reported in the literature on VFA production from food waste,^18^ primary sludge, and organic waste (food wastes, fat and oil, beverage wastes, and dairy wastes).^25^ Alkaline pH promotes the hydrolysis of macromolecular substances and extracellular polymeric substances (EPS) of sludge flocs.^10^ This is because the alkaline pH induces the cleavage of covalent bonds of organic matter to increase the rupture of the cell membrane.^28^ Under alkaline conditions, the charged functional groups in the EPS of digested sludge also get ionized, and the accessibility of the soluble compounds is facilitated.^29^ Further, OH^–^ ions break down peptide bonds for enhanced protein degradation.^30^ Recently, Lin and Li (2018) reported that alkaline pH (8–10) was more effective in VFA production by up to 46% than acidic pH (2–6) due to enzymatic activities.^31^

The VFA composition (Figure 2) suggests that homoacetogenic bacteria and propionic acid-producing bacteria that consume hydrogen are favored at alkaline pH. In addition, the phosphoroclastic pathway anticipated under alkaline pH is also likely the reason for the higher acetate production.^30^ Similar observations of acetate and propionate domination at alkaline conditions were reported in the literature.^25,32^ On the other hand, a higher diversity of volatile and medium chain fatty acids was obtained at acidic conditions compared to alkaline conditions.^27^

#### 4.1.2 VFA Accumulation Due to Inoculum Heat Treatment

VFA accumulation due to heat treatment was more evident under alkaline conditions than under acidic conditions (Figure 1). This could be attributed to the prior inhibition of methanogenesis in an acidic environment, and therefore, heat treatment might have a negligible effect to eliminate methanogenic archaea. Moreover, the acidogenic bacteria can form spores when subjected to high temperatures, whereas methanogens lack that functionality, and therefore, a heat shock at around 80–100 °C for 15–120 min can be employed to deactivate the methanogens.^33^ Sarkar et al. (2016) reported that heat shock resulted in VFA accumulation because it simultaneously selects acidogens and removes methanogens.^34^ Mondylaksita et al. (2021) also reported that heat treatment led to more VFA accumulation than the chemical inhibitor (BES).^33^ The VFA production efficiency could be significantly improved through the simple method of using heat treated inoculum (Figure 1b), although VFA production using inoculum without heat treatment is also well possible.^18^

#### 4.1.3 Simultaneous VFA Production during Selenium Bioremediation

This study shows that food waste, one of the most abundant wastes, can be used for selenium remediation. The mechanism for bioreduction of selenium oxyanions has been well documented.^41^ Several microorganisms can use selenate as a terminal electron acceptor in anaerobic respiration to conserve energy. Selenate is reduced to selenite and then to insoluble elemental selenium in a two-step process known as dissimilatory reduction by anaerobic respiration. Reduction of selenate to selenite is catalyzed by a trimeric molybdoenzyme, SerABC selenate reductase, located in the periplasmic space.^16^ Selenite formed in the periplasmic place is presumed to be transported to the cytoplasm via a sulfate transporter, where selenite is reduced to elemental selenium. Reduction of selenite in the cytoplasm occurs by thiol-mediated reduction as part of a microbial detoxification strategy.^15^ In addition, enzymes such as nitrite reductase, sulfite reductase, fumarate reductase, and hydrogenase I can also support the reduction of selenite to elemental selenium in some microorganisms (such as *Clostridiaceae* reported in this study). The extracellular expulsion of selenium nanospheres produced in the cytoplasm normally occurs during anaerobic dissimilatory reduction.^16^

Although the literature is scarce on biological selenium oxyanion reduction using food waste as the carbon source and electron donor, biological sulfate reduction using food industry waste has been reported.^35^ This study reveals that alkaline pH was more favorable for Se removal than acidic pH (Figure 3). Tan et al. (2018) also reported a higher SeO_4_^2–^ removal efficiency in alkaline conditions; however, lowering the pH to 5 was detrimental to the up-flow anaerobic sludge bed (UASB) reactor performance as the SeO_4_^2–^ removal efficiency dropped by 20–30%.^36^ Additionally, a low polysaccharide (PS) to protein ratio in the soluble extracellular polymeric substance matrix of the biomass at low pH can affect the biomass properties.^36^ A pH value above 5 is a prerequisite for consumption of the carbon source for efficient sulfate reduction.^37^ For instance, Martins et al. (2009) supplemented calcite tailing that acts as a neutralizing and buffer material to increase the pH of food industry waste from 4 to 6 for dissimilatory sulfate reduction.^35^

In this study, the removal of selenite was accomplished earlier than that of selenate (Figure 3). This could be due to the high reactivity of SeO_3_^2–^ to form strong stable bonds with organic matter in the food waste, leading to its easy and fast removal, whereas SeO_4_^2–^ is less reactive and forms weakly bound complexes and is therefore harder to remove.^38,39^ Our previous study on anaerobic codigestion of dissolved air floatation slurry with Se oxyanions containing synthetic wastewater showed that the Se removal was faster in the presence of SeO_3_^2–^ than SeO_4_^2–^.^22^ It should be noted that the effect of Se oxyanions was clearly evident only on and after day 10. This could be because the onset of the exponential phase of methanogenesis is typically with a lag phase of about 10 days for food waste.^40^ Similarly, Mondylaksita et al. (2021) reported acceleration of VFA production after day 10 of fermentation of the glucan-rich fraction of palm oil empty fruit bunch.^33^

This study clearly indicates that heat treatment of the inoculum is detrimental for Se removal in the presence of selenate, especially at acidic pH. It is attributed to the inhibition in the presence of selenium reducing bacterial families after the combination of acid and heat pretreatments, evident from the high-throughput sequencing (as discussed in Section 4.1.4). Further research is required to ascertain the mechanism underpinning the effect of acid and heat treatments on the microbial community composition.

Our earlier study in a UASB reactor treating selenate-rich wastewater showed that repeated exposure to Se oxyanions at higher concentrations led to reactor acidification and impaired methane production.^17^ Especially, the relative abundance of *Methanosaeta* (an acetoclastic methanogenic archaea) decreased from 75% to less than 15% at the RNA level. The toxicity of selenium on methanogenic archaea but less on fermentative bacteria could be leveraged to recover fermentative products such as carboxylic acids and alcohols. The elemental Se nanoparticles synthesized in this study were in the shape of nanospheres (Figure 4), which are typical for mesophilic systems, compared to nanorods synthesized in thermophilic systems.^41^

A commonly used methanogenic inhibitor such as 2-bromoethanesulfonate (BES) is a coenzyme M analog that acts as a competitive inhibitor in methyl transfer reactions.^42^ β-cyclodextrin was also demonstrated as a chemical methanogenic inhibitor to improve hydrogen and VFA production by Kidanu et al. (2017).^43^ However, the market cost of β-cyclodextrin is high (∼ €1250/kg in 2022). Such high costs of external chemical addition might reduce the competitiveness of biobased VFAs over petrochemical-based VFAs, and therefore, it is imperative to identify other cheap inhibitory compounds available in abundance. Bioremediation of selenium-laden wastewaters for VFA production could be a good alternative from the existing chemical inhibitors such as BES and β-cyclodextrin.

#### 4.1.4 Linking Process Performance with Microbial Community Dynamics

The microbial community dynamics was more evident over the retention time at alkaline pH than at acidic pH, similar to their process performance. pH governs the microbial cell structural integrity and metabolism^44^ and influences the occurrence and distribution of microorganisms originating from the same inocula. High temperatures have profound effects on the structural and physiological properties of bacteria, affecting their membranes, RNA, DNA, ribosomes, protein and enzymes.^45^ The bacteria that are sporulating, hyperthermophiles/extremophiles, or with thermophilic spores are less susceptible and extremely resistant to heat.^46^

*Porphyromonadaceae* and *Veillonellaceae* were the dominant bacterial families at alkaline and acidic pH, respectively. *Porphyromonadaceae* is reported to play a central role in fermentation of glucose from food waste.^47^ Similarly, *Clostridiaceae* is known for the degradation of cellulose present in plant-based foods.^48^ Meanwhile, a mixture of fermentation products from a wide range of carbohydrates is produced by *Bacteroidaceae*.^49^*Ruminococcaceae* is closely linked with the production of caproic acid.^50,51^*Prevotellaceae* and *Lachinospiraceae* can be directly associated with the VFA components especially acetate, butyrate, propionate, and caproate, with many species members active in the fermentation of carbohydrates present in the food waste.^52^ Finally, it is also noteworthy that many members of bacterial families such as *Prevotellaceae*, *Ruminococcaceae*, *Clostridiaceae*, and *Lachnospiraceae* are reported to perform homoacetogenesis resulting in the dominant acetic acid production at alkaline pH.^53^

Heat treatment resulted in a significant drop in RA of families such as *Porphyromonadaceae*, *Marinilabiaceae*, *Bacteroidaceae*, and *Prevotellaceae* that host non-spore-forming bacteria. Conversely, most bacteria in families such as *Ruminococcaceae* and *Clostridiaceae* are sporulating and therefore were less susceptible to heat treatment. It is most likely that these heat-resistant bacteria exhibited cell responses such as intracellular heat-shock protein induction and extracellular alarmone activation.^45^ Similarly, the presence of methanogenic archaea, especially *Methanobacteriaceae*, was reduced after the heat treatment in both pH conditions. This indicates that heat treatment of the inoculum is a simple yet effective strategy for methanogenic inhibition but retains acidogenic spore-forming bacteria.

High RAs of the families that host novel selenium-reducing bacteria such as *Tissierellaceae*, *Clostridiaceae*, and *Peptococcaceae* were found in both acidic and alkaline pH in this study.^54−56^ This explains the high selenium removal efficiency under both pH conditions. Notably, the relative abundance of *Prevotellaceae* is reported to have been strengthened in the presence of selenium.^57−59^ Similarly, *Bacteroidaceae* is a well-known selenium-reducing bacterial family.^60^ However, the presence of these families that were found dominant at acidic pH decreased following the heat treatment, which could possibly result in the reduced Se removal rate. It is noteworthy that selenium reducing bacteria might enhance production of VFAs under alkaline pH, similar to sulfur reducing bacteria.^61^ Nevertheless, cDNA-level microbial analyses could better reveal the effect of Se oxyanions, compared to the DNA based communities.^17^

### 4.2 Future Perspectives for Volatile Fatty Acid Production and Selenium Bioremediation

**Figure 7:**
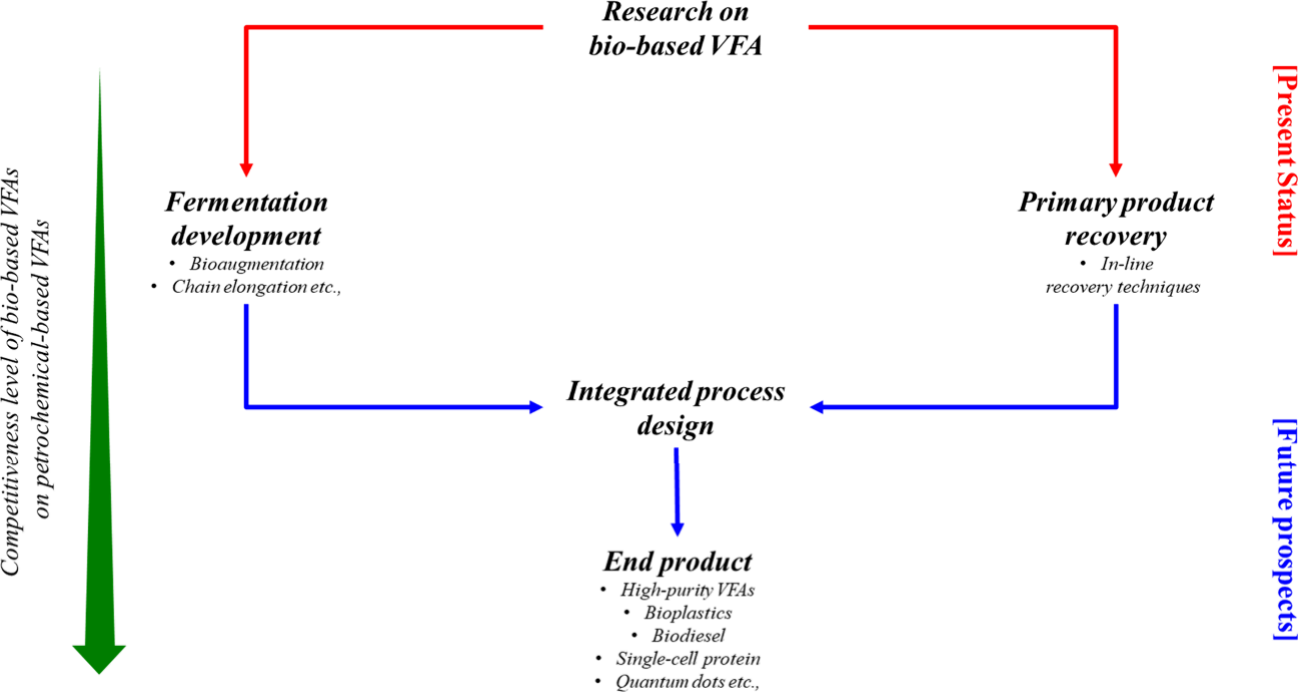
Current status and future prospects of biobased VFA research.

As illustrated in Figure 7, there are two different approaches in the research area on biobased VFAs, viz., (1) fermentation development and (2) primary product separation and recovery. A paradigm shift is needed on isolated approaches toward the development of the end product through integrated process design.^62^ Conventionally, the primary production is optimized through operational conditions, such as inoculum, substrate, pH, temperature, and retention time. More diverse VFA presence in low concentrations discourages businesses from adopting them due to concerns in terms of their recovery. However, biotechnological advances in the past decade have focused on addressing this challenge. Novel strategies are being developed, such as bioaugmentation for targeted tailor-made VFA production.^63^ Recently, the chain elongation process that occurs via the reversed β-oxidation pathway and the fatty acid biosynthesis pathway to produce less hydrophilic medium chain carboxylic acids (C_6_–C_12_) through secondary fermentation of short-chain organic acids has gained interest as well.^51^ VFAs can be developed for end-use applications such as alcohol, bioplastics, biodiesel, biological nutrient recovery, and electricity generation.^9^ Presently, the recovery methods include gas stripping with absorption, adsorption, solvent extraction, electrodialysis, reverse osmosis, nanofiltration, and membrane contractor;^6^ however, the prospects are unfolding for in-line recovery.^64^ Recently, research on novel online monitoring of VFAs in anaerobic digesters has also been receiving attention.^65^

Because research on anaerobic fermentation has high degrees of nonuniformity (from feedstocks to inoculum microbiome), technologies such as metagenomics and metatranscriptomic analysis would provide insights for generalized conclusions. Considering that these applications have huge commercial implications, several industries in a varied range of spectra will leverage the research and developments on biobased VFA production. This study indicates the feasibility of anaerobic treatment of acidic Se-laden wastewater such as mining wastewater in a single-stage bioreactor for VFA and Se nanoparticle production without any additional cost of pretreatment for neutralization.

This study also implies that biological resource recovery could be concomitantly achieved during the anaerobic fermentation process for VFA production. This batch work necessitates that a long-term continuous bioreactor performance for simultaneous VFA and elemental Se nanoparticle production should be demonstrated. Additionally, a technoeconomic and life cycle assessment will be beneficial for commercial implementation. Se levels in industrial wastewaters are higher (>100 μM) and are reported to inhibit methanogenesis. Nanchariah and Lens (2015) also stated that VFAs produced from AD of food waste could serve as an electron donor for Se reduction.^16^ Hence, remediation of Se wastewater could be achieved in a single-stage bioreactor, producing volatile fatty acids. Nonetheless, whenever Se regulatory discharge limits could not be met, additional post-treatment methods should be employed.

## 5 Conclusions

The effects of pH, inoculum heat treatment, and selenium supplementation on VFA production, Se removal, and microbial community structure using food waste as the carbon source were investigated. An improved VFA yield (9315 ± 652 mg COD/L) was achieved at alkaline pH, which was 45% higher than that of acidic pH. Selenium was shown to be a chemical inhibitor to accumulate VFAs (8967 ± 569 mg COD/L) at alkaline pH with non-heat treated inoculum. Furthermore, heat treatment was effective in VFA accumulation (10241 ± 430 mg COD/L) only at alkaline pH. Acetate and propionate were the dominant VFAs produced in alkaline condition without heat treatment; however, a diversified VFA composition was observed at all other conditions tested. More than 95% selenium removal was accomplished at the tested conditions, except at acidic pH with heat treatment due to reduced relative abundance of selenium-reducing bacterial families (such as *Bacteroidaceae* and *Prevotellaceae*).

## Supporting Information Available

Experimental sets conducted, initial VFAs in food waste, VFA production and Se removal at 100 and 300 μM Se oxyanions, pH profiles, and principal component analysis of microbial community (PDF)

## ABBREVIATIONS

AD: anaerobic digestion
APHA: American Public Health Association
BES: 2-bromoethanesulfonic acid
BOD: biological oxygen demand
DNA: deoxyribonucleic acid
COD: chemical oxygen demand
EDX: energy dispersive X-ray spectroscopy
eq: equation
EU: European Union
GHG: greenhouse gas
HRT: hydraulic retention time
HT: heat treated inoculum
LCFA: long-chain fatty acid
NHT: non-heat treated inoculum
OLR: organic loading rate
PCA: principal component analysis
*p*value: probability value
RA: relative abundance
SEM: scanning electron microscopy
SeO_4_^2–^: selenate
SeO_3_^2–^: selenite
TEM: transmission electron microscopy
TS: total solid
VFA: volatile fatty acid
VS: volatile solid
