# Supplementary material for: Influence of pH, Heat Treatment of Inoculum, and Selenium Oxyanions on Concomitant Selenium Bioremediation and Volatile Fatty Acid Production from Food Waste
Source: ACS Omega. 2023 Sep 14;8(38):34397–409. doi: 10.1021/acsomega.2c06459 (PMC10535259; doi:10.1021/acsomega.2c06459)
Supplement: Supplementary file 1 — ao2c06459_si_001.pdf [file ao2c06459_si_001.pdf]

Supplementary Information

Influence of pH, heat treatment of inoculum and selenium oxyanions on concomitant selenium bio-remediation and volatile fatty acids production from food waste

Mohanakrishnan Logan [a,b], Fengyi Zhu [c], Piet N. L. Lens [b], Zeynep Cetecioglu [a,c]*

[a] Department of Chemical Engineering, School of Engineering Sciences in Chemistry, Biotechnology and Health, KTH Royal Institute of Technology, SE100 44, Stockholm, Sweden

[b] Department of Microbiology, School of Natural Sciences and Ryan Institute, National University of Ireland, University Road, H91 TK33, Galway, Ireland

[c] Department of Industrial Biotechnology, School of Engineering Sciences in Chemistry, Biotechnology and Health, KTH Royal Institute of Technology, SE106 91, Stockholm, Sweden

Corresponding Author

* Mohanakrishnan Logan (mohanlogan@iitm.ac.in)

Department of Chemical Engineering, School of Engineering Sciences in Chemistry, Biotechnology and Health, KTH Royal Institute of Technology, SE100 44, Stockholm, Sweden; Department of Microbiology, School of Natural Sciences and Ryan Institute, National University of Ireland, University Road, H91 TK33, Galway, Ireland

Table S1. Experimental sets conducted in this study

**Experimental sets**	**pH**	**Inoculum**	**Conditions**
I	5	NHT	Control<br>100, 300 and 500 µM SeO_4^{2-}<br>100, 300 and 500 µM SeO_3^{2-}
II	10	NHT	Control<br>100, 300 and 500 µM SeO_4^{2-}<br>100, 300 and 500 µM SeO_3^{2-}
III	5	HT	Control<br>500 µM SeO_4^{2-}<br>500 µM SeO_3^{2-}
IV	10	HT	Control<br>500 µM SeO_4^{2-}<br>500 µM SeO_3^{2-}

HT – heat treated inoculum; NHT – non-heat treated inoculum

Table S2. Initial average volatile fatty acid concentration (in mg COD/L) introduced into batch reactors from food waste

**Acetate**	**Propionate**	**Isobutyrate**	**Butyrate**	**Isovalerate**	**Valerate**	**Isocaproate**	**Caproate**	**Total VFA**
1365	206	749	128	187	34	-	-	2669

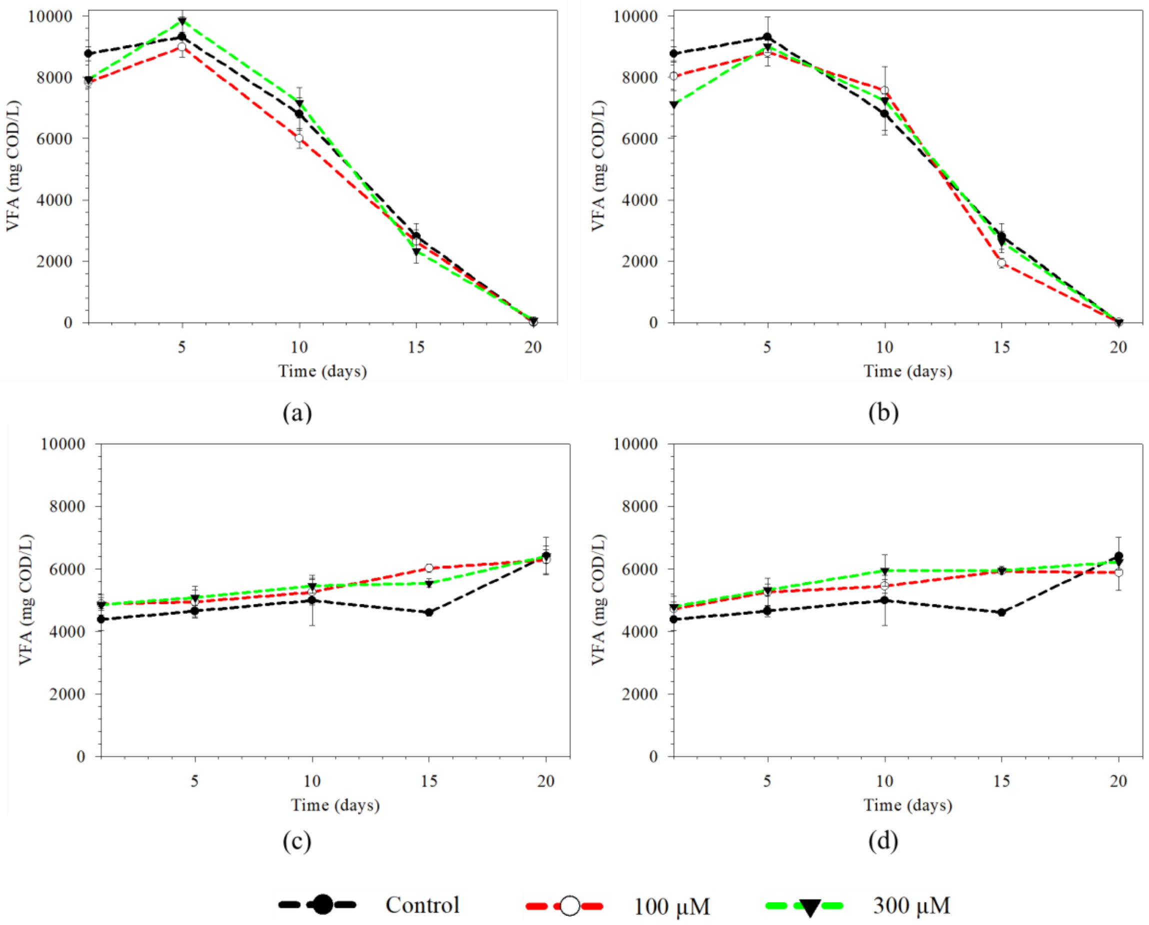

Figure S1. Volatile fatty acids profile at (a) pH 10, NHT, SeO_4^{2-}; (b) pH 10, HT, SeO_3^{2-}; (c) pH 5, NHT, SeO_4^{2-}; and (d) pH 10, HT, SeO_4^{2-}. NHT and HT stands for non-heat treated and heat treated inoculum, respectively.

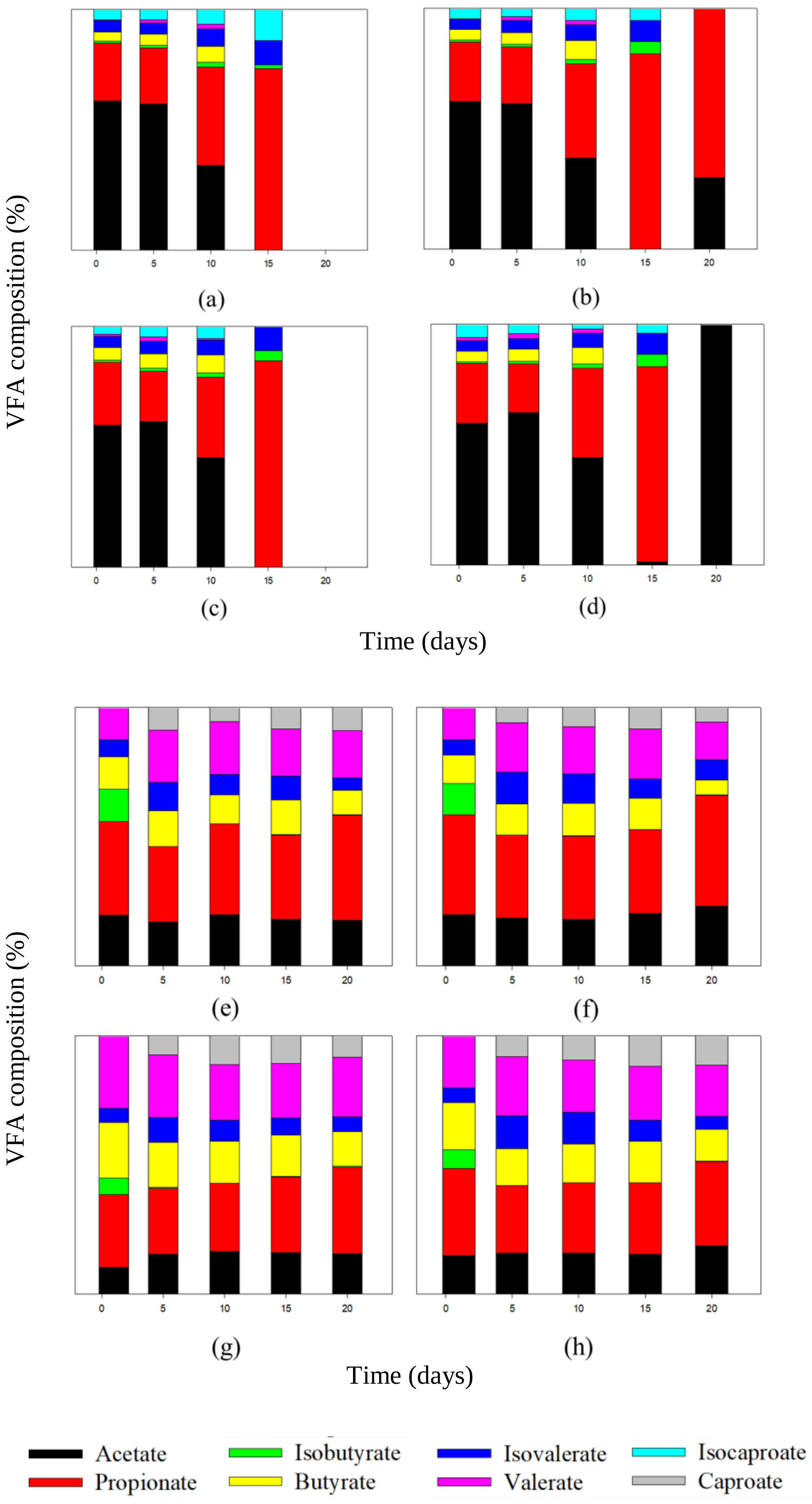

Figure S2. Volatile fatty acids composition with non-heat treated inoculum at (a) pH 10, 100 μM SeO_4^{2-}; (b) pH 10, 300 μM SeO_4^{2-}; (c) pH 10, 100 μM SeO_3^{2-}; (d) pH 10, 300 μM SeO_4^{2-}; (e) pH 5, 100 μM SeO_4^{2-} ; (f) pH 5, 300 μM SeO_4^{2-}; (g) pH 5, 100 μM SeO_3^{2-}; and (h) pH 5, 300 μM SeO_4^{2-}. For abbreviations, see Figure S1.

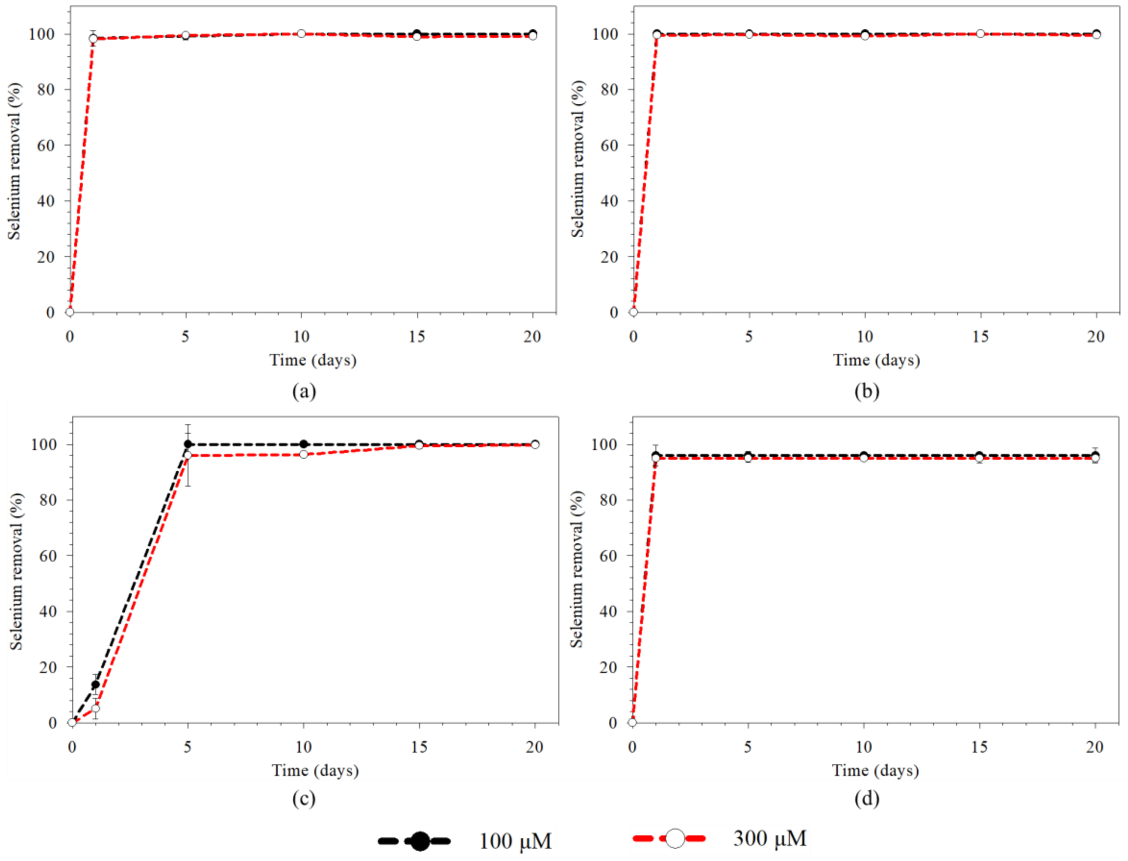

Figure S3. Selenium removal with non-heat treated inoculum at (a) pH 10, SeO_4^{2-}; (b) pH 10, SeO_3^{2-}; (c) pH 5, SeO_4^{2-}; and (d) pH 5, SeO_3^{2-}

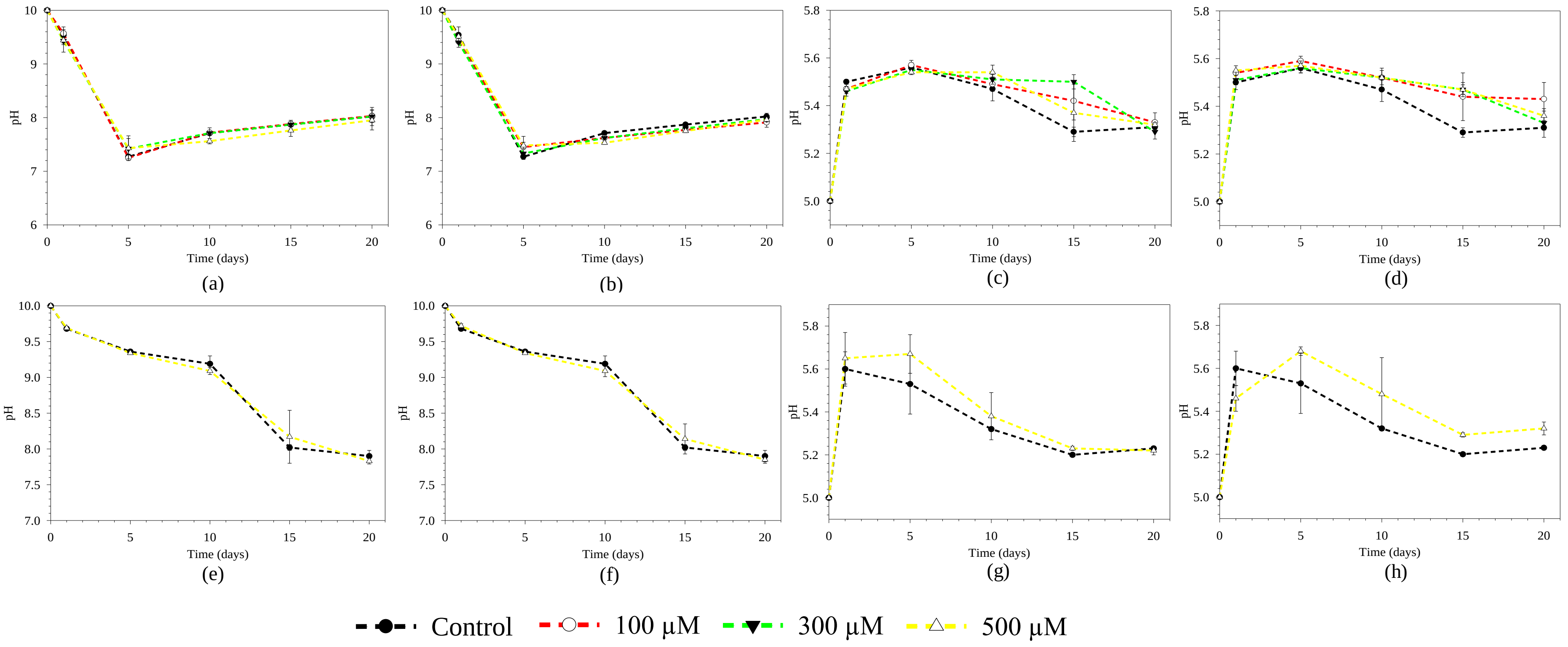

Figure S4. pH variation with non-heat treated inoculum at (a) pH 10, SeO_4^{2-}; (b) pH 10, SeO_3^{2-}; (c) pH 5, SeO_4^{2-}; and (d) pH 5, SeO_3^{2-}; and heat treated inoculum at (e) pH 10, SeO_4^{2-}; (f) pH 10, SeO_3^{2-}; (g) pH 5, SeO_4^{2-}; and (h) pH 5, SeO_3^{2-}

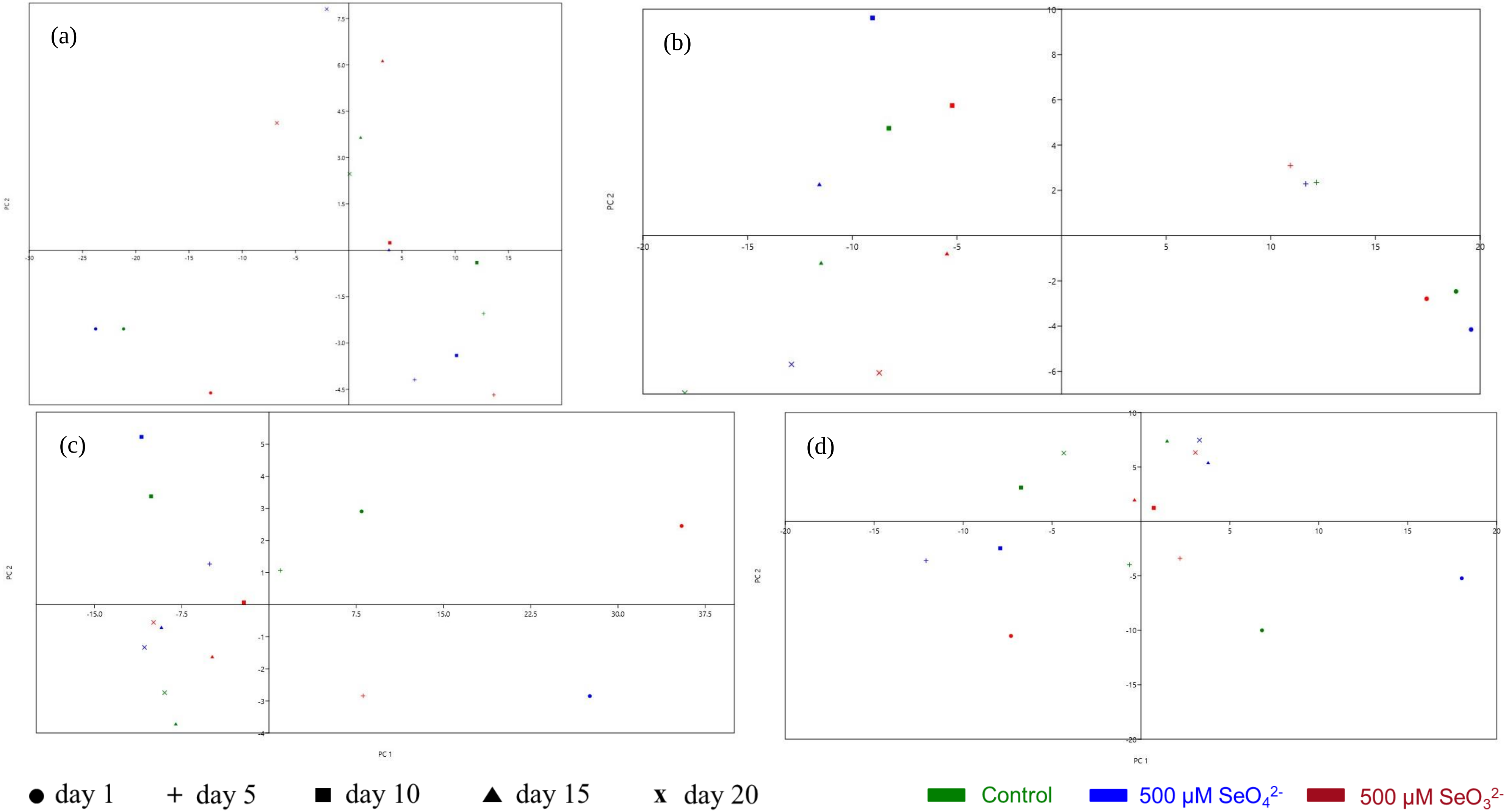

Figure S5. Principal Component Analysis of the microbial community on family level for (a) pH 10 – NHT; (b) pH 10 – HT; (c) pH 5 – NHT; and (d) pH 5 – HT. For abbreviations, see Figure S1.
